# Fluorescent/phosphorescent dual-emissive conjugated polymer dots for hypoxia bioimaging†
†Electronic supplementary information (ESI) available. See DOI: 10.1039/c4sc03062a



**DOI:** 10.1039/c4sc03062a

**Published:** 2015-01-12

**Authors:** Qiang Zhao, Xiaobo Zhou, Tianye Cao, Kenneth Yin Zhang, Lijuan Yang, Shujuan Liu, Hua Liang, Huiran Yang, Fuyou Li, Wei Huang

**Affiliations:** a Key Laboratory for Organic Electronics and Information Displays & Institute of Advanced Materials (IAM) , Jiangsu National Synergistic Innovation Center for Advanced Materials (SICAM) , Nanjing University of Posts & Telecommunications , 9 Wenyuan Road , Nanjing 210023 , China . Email: iamqzhao@njupt.edu.cn ; Email: wei-huang@njtech.edu.cn ; Fax: +86-25-85866396; b Key Laboratory of Flexible Electronics (KLOFE) & Institute of Advanced Materials (IAM) , Jiangsu National Synergistic Innovation Center for Advanced Materials (SICAM) , Nanjing Tech University (NanjingTech) , 30 South Puzhu Road , Nanjing 211816 , China; c Department of Chemistry & State Key Laboratory of Molecular Engineering of Polymers & Institute of Biomedicine Science , Fudan University , Shanghai 200433 , China . Email: fyli@fudan.edu.cn ; Fax: +86-21-55664621

## Abstract

A kind of fluorescent/phosphorescent dual-emissive conjugated polyelectrolyte has been prepared by introducing phosphorescent platinum(ii) porphyrin (O_2_-sensitive) into a fluorene-based conjugated polyelectrolyte (O_2_-insensitive), which can form ultrasmall conjugated polymer dots (FP-Pdots) in the phosphate buffer solution (PBS) *via* self-assembly caused by their amphiphilic structures with hydrophobic backbones and hydrophilic side chains. These FP-Pdots can exhibit an excellent ratiometric luminescence response to O_2_ content with high reliability and full reversibility for measuring oxygen levels, and the excellent intracellular ratiometric O_2_ sensing properties of the FP-Pdots nanoprobe have also been confirmed by the evident change in the *I*_red_/*I*_blue_ ratio values in living cells cultured at different O_2_ concentrations. To confirm the reliability of the O_2_ sensing measurements of the FP-Pdots nanoprobe, O_2_ quenching experiments based on lifetime measurements of phosphorescence from Pt(ii) porphyrin moieties have also been carried out. Utilizing the sensitivity of the long phosphorescence lifetime from Pt(ii) porphyrins to oxygen, the FP-Pdots have been successfully applied in time-resolved luminescence imaging of intracellular O_2_ levels, including photoluminescence lifetime imaging and time-gated luminescence imaging, which will evidently improve the sensing sensitivity and reliability. Finally, *in vivo* oxygen sensing experiments were successfully performed by luminescence imaging of tumor hypoxia in nude mice.

## Abstract 2

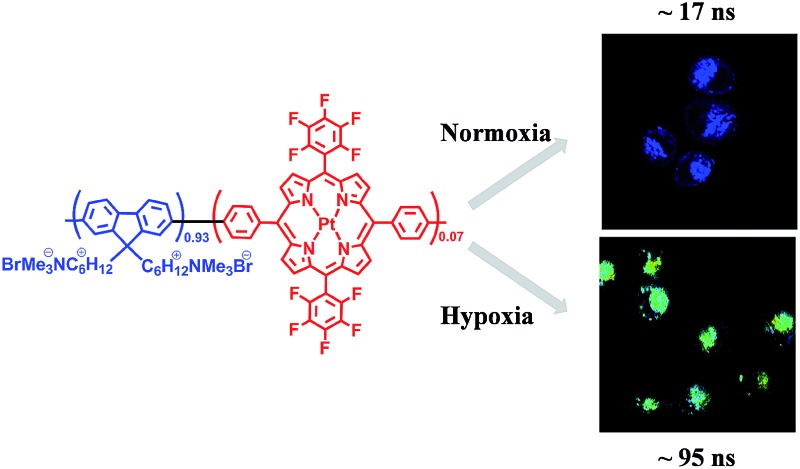
Fluorescent/phosphorescent dual-emissive conjugated polymer dots were designed and synthesized, ere used for tumor hypoxia sensing *via* ratiometric imaging and photoluminescence lifetime imaging.

## Introduction

Hypoxia has been found to be closely related to various diseases, such as solid tumors,^1^ brain abnormalities^2^ and retinal diseases.^3^ Especially, hypoxia is a feature of tumor tissues, and the median oxygen (O_2_) concentration in some solid tumors is around 4% and may even decrease to 0% locally.^4^ The real-time monitoring of O_2_ concentration in living cells and tissue can not only lead to accurate diagnosis of cancer, but can also be used to evaluate therapeutic effects. Thus far, enormous efforts have been focused on the development of hypoxia sensing. Hypoxia can be selectively detected by utilizing immunostaining,^5^ magnetic resonance imaging,^6^ positron emission tomography imaging,^7^ and optical imaging techniques.^8–10^ Among them, non-invasive optical imaging offers a powerful approach to map oxygen in living cells and tissues with high sensitivity and spatial resolution. Many fluorescence probes for optical imaging that employ organic dyes containing a nitro group, quinone group, or azo group as the hypoxia-sensing moiety have been developed.^10^ Most of these probes, however, usually show an irreversible fluorescence change upon bioreduction reaction under hypoxia, which limits their applications in real-time fluorescence monitoring of oxygen concentration.

Phosphorescent probes based on transition-metal complexes can be used for fully reversible real-time monitoring of O_2_ levels *in vitro* with high-resolution by utilizing the energy transfer between the triplet excited state of the metal complex and the triplet ground state of O_2_.^8,11^ Thus, phosphorescent metal complexes with a long-lived triplet excited state, such as platinum(ii),^12^ palladium(ii),^13^ ruthenium(ii),^14^ and iridium(iii)^15^ complexes, have been regarded as a kind of fascinating hypoxia probe. The phosphorescence intensities of these complexes are reversibly dependent on the change in O_2_ concentration. However, most of these reported probes are based on the variation in single phosphorescent emission (“ON–OFF” or “OFF–ON” type). The single intensity-based reporting signal is easily influenced by the external environment, such as the probe's concentration, temperature, or pH value. The standardization and use of accurate readout of O_2_ concentration are also difficult. Therefore, an advisable choice is to develop ratiometric O_2_ probes that can allow for accurate measurement of O_2_ concentration through the ratio change of the emission intensities at two different wavelengths.

**Fig. 1:**
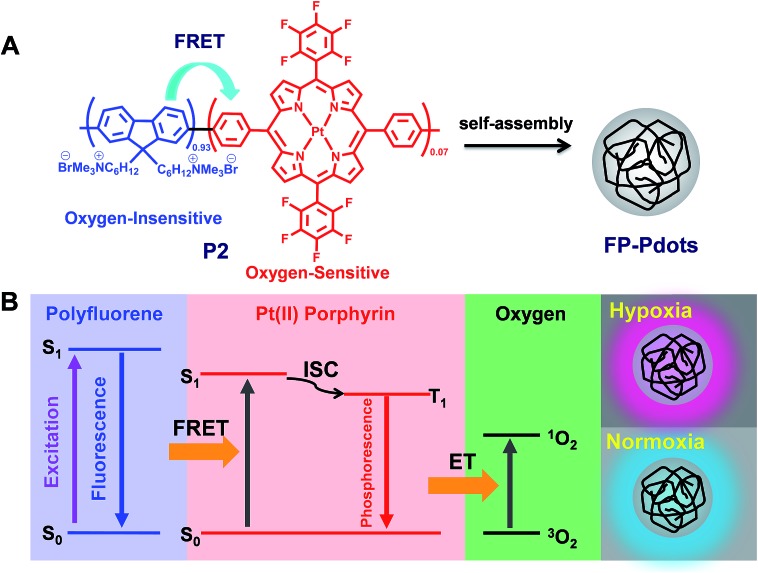
(A) The chemical structure of fluorescent/phosphorescent conjugated polyelectrolyte **P2**, and a schematic illustration of the self-assembly behavior of **P2** into the polymer dots (FP-Pdots); (B) oxygen sensing mechanism of conjugated polyelectrolyte and schematic illustrations of the energy level of the moieties in FP-Pdots.

For the design of ratiometric O_2_ probes, herein, we hope to demonstrate an effective way to construct a Förster resonance energy transfer (FRET) system with an O_2_-insensitive fluorophore as the energy donor and an O_2_-sensitive phosphorescent metal complex as the acceptor (Fig. 1). The occurrence of FRET can significantly improve the sensitivity of sensing by promoting the population of the oxygen-sensitive triplet excited state of the acceptor. We introduced phosphorescent platinum(ii) porphyrin (O_2_-sensitive) into a fluorene-based conjugated polyelectrolyte (O_2_-insensitive). Fluorescent/phosphorescent dual-emissive polymer dots (FP-Pdots, see Fig. 1A) were successfully prepared as excellent O_2_ probes. The as-prepared FP-Pdots enabled ratiometric measurements of hypoxia in living cells with the advantages of eliminating photobleaching and excitation power fluctuation by using the O_2_-insensitive fluorescence from polyfluorene moieties as a reference and O_2_-sensitive phosphorescence from Pt(ii) porphyrins as the sensing signal.^16^ Taking advantage of the long excited-state lifetime of phosphorescent Pt(ii) porphyrins, time-resolved luminescence imaging of O_2_ levels in living cells, including photoluminescence lifetime imaging microscopy (PLIM) and time-gated luminescence imaging (TGLI), was realized. These techniques can eliminate external influences and autofluorescence interferences from biological samples.^17,18^ Furthermore, we applied the FP-Pdots probe to nude mice for the luminescence imaging of tumor hypoxia. These experiments have shown that FP-Pdots are an excellent class of luminescent probes for practical application in determining O_2_ concentrations in living samples.

## Results and discussion

### Design, synthesis and properties of the FP-Pdots

**Scheme 1:**
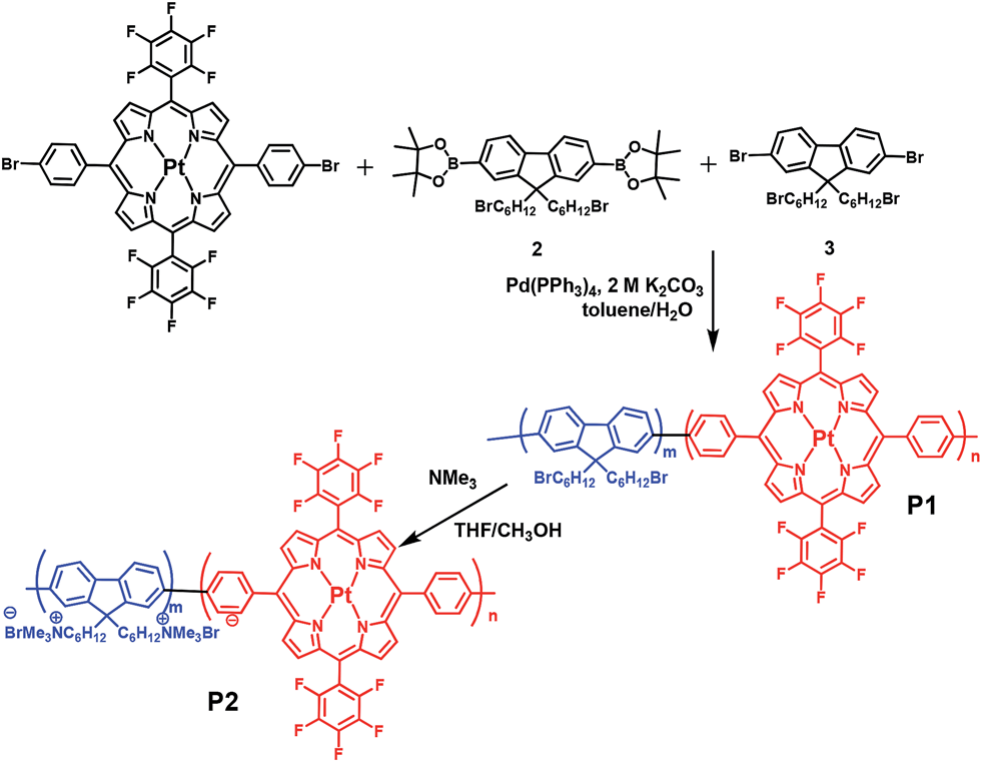
Synthetic route of the target conjugated Pt(ii) complex–polyelectrolyte **P2** and the intermediate **P1**.

We chose conjugated polymer as the oxygen-insensitive energy donor to construct FRET-based ratiometric O_2_ probes (Fig. 1) because of their high light absorptivity, extraordinary fluorescence brightness, excellent photostability, and good water-dispersibility.^19,20^ The synthetic route and chemical structures of the conjugated polyfluorene electrolyte are shown in Scheme 1. The Pt(ii) complex–polymer precursor **P1** was prepared *via* a Suzuki polycondensation reaction, and its structure was characterized *via* ^1^H NMR and ^13^C NMR. The number-average molecular weight of the polymer was determined to be 25 400 with a polydispersity index of 2.49, measured *via* gel permeation chromatography (GPC) in THF by using the calibration curve of polystyrene standards. The target Pt(ii) complex–polymer **P2** was obtained by the quarternization of the precursor **P1** (Scheme 1). The initial feed ratio of Pt(ii) porphyrins to the total monomers was 10 mol%. The actual Pt(ii) porphyrin content in the copolymer, which was estimated *via* ^1^H NMR, was about 7 mol%. This value is lower than that of the feed ratio probably because of the reaction activity or steric hindrance.

**Fig. 2:**
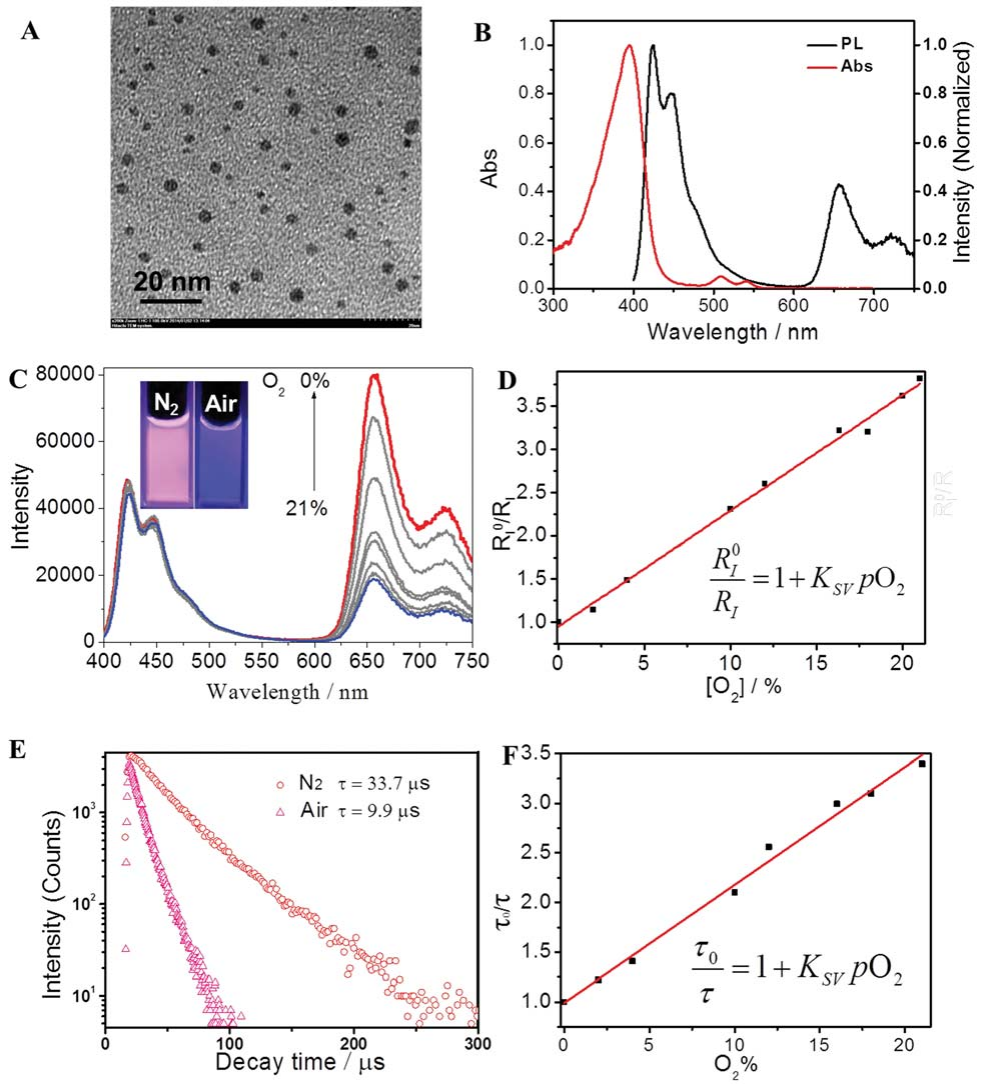
(A) A TEM image of FP-Pdots in aqueous solution; (B) normalized absorption and emission spectra of FP-Pdots (12 μg mL^–1^) in aqueous solution; (C) emission spectra of FP-Pdots (12 μg mL^–1^) in H_2_O under the different O_2_ concentrations; inset: the emission photos of FP-Pdots in the presence and absence of O_2_; *λ*_ex_ = 375 nm. (D) Plot of *R*_*I*_^0^/*R*_*I*_ as a function of O_2_ concentration; (E) phosphorescence decays of FP-Pdots in aqueous solution saturated with N_2_ or air, monitored at 656 nm from the Pt(ii) porphyrin moiety; *λ*_ex_ = 405 nm. (F) Plot of *τ*_0_/*τ* as a function of O_2_ concentration (*λ*_ex_ = 405 nm).

The morphology of **P2** in the phosphate buffer solution (PBS) was investigated *via* transmission electron microscopy (TEM) and dynamic light scattering (DLS), as shown in Fig. 2A and S1.† Each of the FP-Pdots have a diameter of approximately 5 nm and they were formed *via* self-assembly caused by their amphiphilic structures with hydrophobic backbones and hydrophilic side chains. Thus, the as-prepared FP-Pdots were well dispersed in PBS. In addition, the zeta-potential of **P2** was measured to be 54.00 mV, which further demonstrated the good dispersibility and stability of the FP-Pdots in PBS.

A particular degree of spectral overlap between the emission spectrum of Pt(ii)-free polyfluorene **P3** (see Scheme S1†) and the absorption spectrum of Pt(ii) porphyrin ensures the efficient energy transfer from polyfluorene segments as host to Pt(ii) porphyrin moieties as guest (Fig. S4†). The photophysical properties of **P2**, specifically of the FP-Pdots, are shown in Fig. 2B. The UV-vis absorption spectrum of the FP-Pdots is dominated by a strong featureless transition centered at 395 nm, which consists of a mixture of Soret bands of Pt(ii) porphyrin^21^ and the π–π* transition of fluorene units. An additional two weak and broad absorptions, which peaked at 510 and 540 nm, respectively, were also found. These results correspond to Q(1,0) and Q(0,0) of the Pt(ii) porphyrin moieties.^21^ FP-Pdots excited at 375 nm exhibit strong blue fluorescence, attributed to the π–π* emission of the fluorene moieties, as well as relatively weak red phosphorescence of the Pt(ii) porphyrin moieties (Fig. 2B). In addition, the FP-Pdots of **P2** also show more efficient FRET than the blend of Pt(ii) porphyrin (**1**) and Pt(ii)-free polyelectrolyte **P3** according to the PL spectra (Fig. S5†). Although the mixture of **1** and **P3** showed the same molar ratio of Pt(ii) porphyrin and fluorene to **P2**, the emission from the Pt(ii) porphyrin was weaker than that in **P2**. The Förster radius is calculated to be 4.7 nm,^15*a*^ demonstrating the effectiveness of covalent incorporation of Pt(ii) porphyrin into **P2**. It also indicated that the dose of Pt(ii) porphyrin was reduced when FRET was present. Therefore, the nanoprobe will be less toxic to cells and animals. Furthermore, due to the high molar absorption coefficient and quantum yield of polyfluorene, the FRET from polyfluorene to Pt(ii) porphyrin will be of benefit for better O_2_-sensitivity than for non-FRET systems.

### Luminescence response to O_2_ content

The PL spectra of the FP-Pdots at various O_2_ concentrations in water at room temperature are shown in Fig. 2C. Under an atmosphere of 21% O_2_, the FP-Pdots showed strong emission centered at 425 nm with a shoulder peak at 450 nm attributed to polyfluorene moieties as well as weak emission at 656 nm with a shoulder peak at 721 nm from Pt(ii) porphyrins. The solution exhibited very bright blue emission under UV excitation (Fig. 2C, inset). With decreasing O_2_ content, the blue fluorescence intensities showed little change, but the red phosphorescence intensities grew progressively. This result is in agreement with the O_2_ sensitivity of the Pt(ii) porphyrin complexes. The red phosphorescence became dominant under an atmosphere of 0% O_2_. Thus, the color of the emission changed from very bright blue to red under UV excitation (Fig. 2C, inset).

The ratiometric oxygen sensing of the FP-Pdots was analyzed quantitatively based on the data of phosphorescence intensities shown in Fig. 2C, according to the Stern–Volmer equation:^22^
1
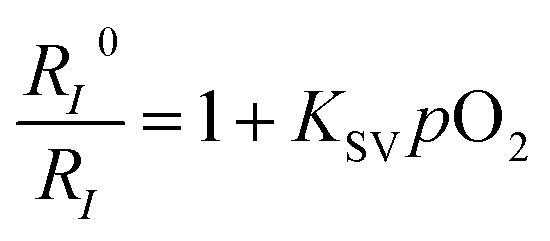
where *K*_sv_ is the Stern–Volmer constant, *p*O_2_ is the oxygen partial pressure, *R*_*I*_^0^ = (*I*_656_^0^/*I*_422_^0^) and *R*_*I*_ = (*I*_656_/*I*_422_) represent the ratios of the phosphorescence (656 nm) intensity of Pt(ii) porphyrin to the fluorescence (422 nm) intensity of the polyfluorene moieties in the absence and presence of O_2_, respectively.

A good linearity of *R*_*I*_^0^/*R*_*I*_ as a function of O_2_ concentration was observed (Fig. 2D and S6†), according to eqn (1). The *K*_sv_ value is 1.67 × 10^–2^ mmHg^–1^. At the same time, no change in the fluorescence intensities of the FP-Pdots monitored at 425 nm from the polyfluorene moiety was observed at various O_2_ concentrations (Fig. 2C), which means that the ratio of the phosphorescence intensity at 656 nm to the fluorescence intensity at 422 nm for the FP-Pdots is dependent on O_2_ concentration. Therefore, the FP-Pdots can be used as a ratiometric phosphorescent probe with high reliability for measuring oxygen levels. The limit of detection of the FP-Pdots was determined to be 0.5 mmHg.

### Lifetime response to O_2_

To confirm the reliability of the O_2_ sensing measurements of the FP-Pdots nanoprobe, we performed similar O_2_ quenching experiments based on lifetime measurements of phosphorescence from Pt(ii) porphyrin moieties, according to eqn (2):2
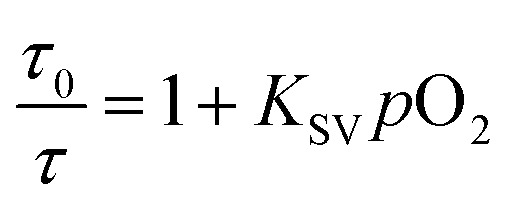
where *K*_sv_ is the Stern–Volmer constant, *p*O_2_ is the oxygen partial pressure, and *τ*_0_ and *τ* are the phosphorescence lifetimes in the absence and presence of O_2_, respectively.

The lifetime (*τ*) monitored at 656 nm decreased evidently with increasing O_2_ concentration, as shown in Fig. 2E. For example, the phosphorescence lifetimes of the FP-Pdots in N_2_ and air were 33.7 and 9.9 μs, respectively. As illustrated in Fig. 2F, a good linear relationship between *τ*_0_/*τ* and *p*O_2_ was observed. The constant *K*_sv_ based on the excited state lifetime was 1.57 × 10^–2^ mmHg^–1^, which is very close to that (1.54 × 10^–2^ mmHg^–1^) obtained from luminescence intensity measurements. However, the fluorescence lifetime of the FP-Pdots monitored at 425 nm from the polyfluorene moiety remains constant at various O_2_ concentrations (Fig. S7†). Therefore, the FP-Pdots can be used as a lifetime-based phosphorescent probe with high reliability for measuring oxygen levels.

### Ratiometric imaging of intracellular O_2_ levels

**Fig. 3:**
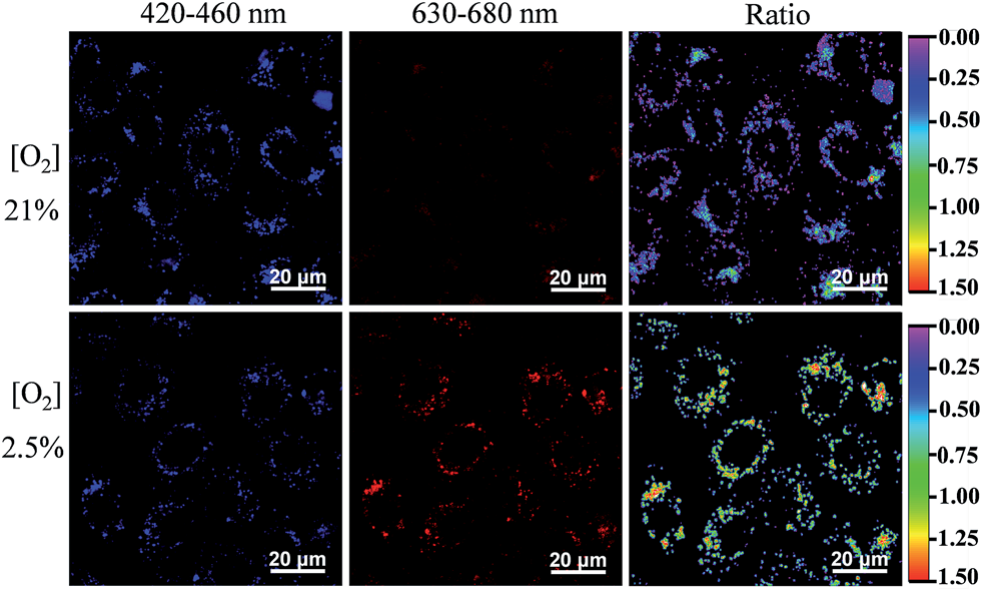
Confocal luminescence imaging and ratiometric luminescence imaging (*λ*_ex_ = 405 nm) of HepG2 cells incubated with the FP-Pdots (10 μg mL^–1^) at 21% or 2.5% O_2_ concentrations. In luminescence imaging, the emission channels at wavelengths of 420–460 nm and 630–680 nm were collected. In ratiometric imaging, the ratio of emission intensity at 630–680 nm to that at 420–460 nm was chosen as the detected signal.

We then used the FP-Pdots as a ratiometric luminescence nanoprobe to investigate intracellular O_2_ levels. The cells were cultured for 24 h at 37 °C at 21% and 2.5% O_2_ concentrations. The excitation wavelength was 405 nm. As shown in Fig. 3, the emission intensities collected at wavelengths from 420 nm to 460 nm (*I*_blue_), which correspond to the reference fluorescence of polyfluorene moieties, are almost the same at 21% and 2.5% O_2_ concentrations. By contrast, the emission images taken in the wavelength range of 630 nm to 680 nm (*I*_red_), which corresponds to the phosphorescence of Pt(ii) porphyrin complexes, show a much brighter image at a condition of 2.5% O_2_ than at 21% O_2_. The excellent intracellular ratiometric O_2_ sensing properties of the FP-Pdots nanoprobe can also be confirmed by the evident change in the *I*_red_/*I*_blue_ ratio values in living cells cultured at different O_2_ concentrations, as shown in ratio images in Fig. 3.

### Time-resolved luminescence imaging of intracellular O_2_ levels

**Fig. 4:**
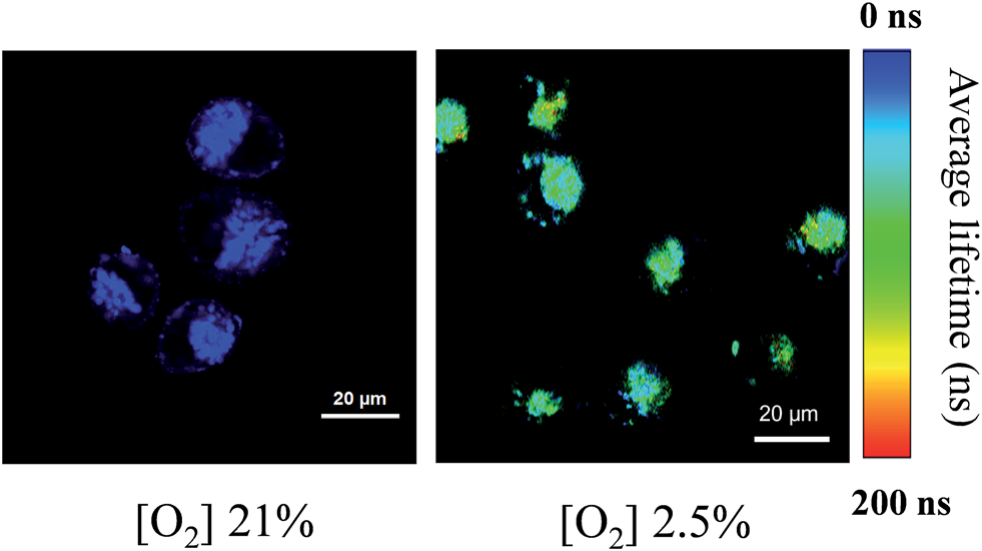
Photoluminescence lifetime images (*λ*_ex_ = 405 nm) of HepG2 cells incubated with 10 μg mL^–1^ FP-Pdots at 37 °C for 2 h at 21% and 2.5% O_2_ concentrations, respectively. The magnification of the objective lens is 40×. The luminescence signals were collected in the range of 420–680 nm.

Considering the response of long phosphorescence lifetime from Pt(ii) porphyrins to oxygen, we have applied our FP-Pdots in time-resolved luminescence imaging of intracellular O_2_ levels. First, the PLIM of intracellular O_2_ level was performed using the FP-Pdots as a lifetime-based nanoprobe, as shown in Fig. 4. The average emission lifetime *τ* was about 17 ns at 21% O_2_ concentration and increased to about 95 ns when the O_2_ concentration was lowered to 2.5%. The interference from short-lived autofluorescence was eliminated because of the relatively long average lifetime of FP-Pdots. This result demonstrated that the FP-Pdots probe exhibits an evident difference in emission lifetime at different O_2_ concentrations and it can be applied as an excellent PLIM probe for sensing intracellular O_2_ levels.

**Fig. 5:**
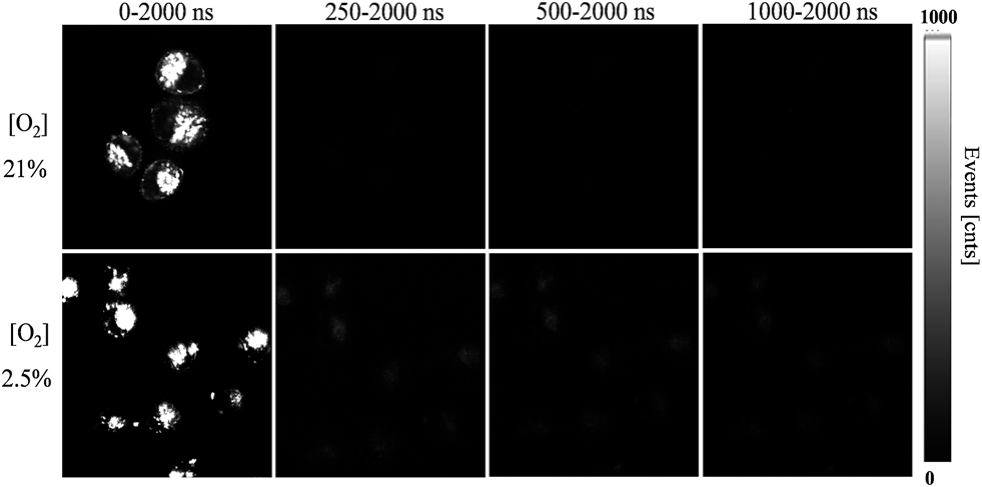
Time-gated luminescence intensity images (*λ*_ex_ = 405 nm) of HepG2 cells incubated with the FP-Pdots (10 μg mL^–1^) at 37 °C for 2 h at 21% or 2.5% O_2_ conditions with different time delays. The magnification of the objective lens is 40×. The luminescence signals were collected in the range of 420–680 nm.

To further demonstrate the ability of anti-interference when the FP-Pdots were applied for O_2_ sensing in living cells, we performed TGLI measurements, and the luminescence intensity images at different time ranges were collected (Fig. 5). When the signal was collected at a time range of 0 ns to 2000 ns, the images of FP-Pdot-treated HepG2 cells exhibited a high signal intensity at 21% and 2.5% O_2_ concentrations because of the presence of reference fluorescence from polyfluorene moieties. Once a particular time delay was exerted, such as in the image collected at a time range of 250 ns to 2000 ns, the signal intensity of the FP-Pdot-treated HepG2 cells at 21% O_2_ concentration could not be observed. By contrast, the signal intensity collected at a time range of 250 ns to 2000 ns at 2.5% O_2_ concentration was still high enough to be observed, although the decrease in the luminescence intensity of the FP-Pdot-treated HepG2 cells is reasonable. When the time delay was increased to 500 ns, the TGLI image of the FP-Pdot-treated HepG2 cells can still be measured. This phenomenon is attributed to the long phosphorescence lifetime of the FP-Pdots. The difference in the emission intensity at O_2_ concentrations of 21% and 2.5% became increasingly more evident, which indicated that O_2_ sensing can be made more sensitive by collecting the signal at a long time range *via* the TGLI technique. As far as we know, this study is the first to use Pdots-based TGLI for intracellular oxygen detection.

### Luminescence imaging of tumor hypoxia in nude mice

**Fig. 6:**
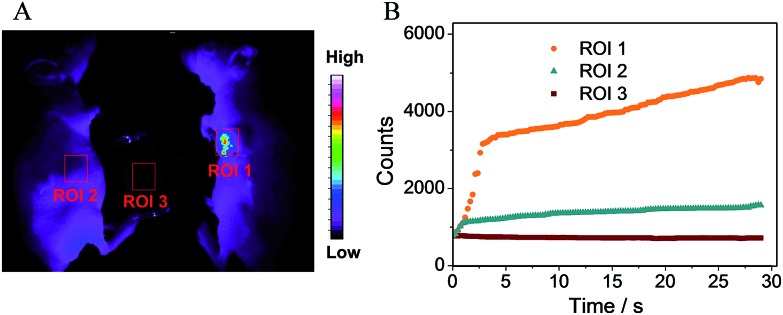
(A) Luminescence imaging *in vivo* of a tumor-bearing mouse (ROI 1) and the control nude mouse (ROI 2) after injection of the FP-Pdots. (B) The change in luminescence intensity of the marked regions (ROI 1, ROI 2 and ROI 3) following different times after injection of the FP-Pdots into the nude mice. A xenon lamp was used as the excitation source, collocating with a bandpass filter (410 ± 15 nm).

To further confirm the potential of the probe for practical application, we applied the FP-Pdots probe for luminescence imaging of tumor hypoxia in nude mice. *In vivo* and *ex vivo* luminescence imaging were performed using a modified Kodak *in vivo* imaging system. The luminescence signals were collected at 660 ± 13 nm. The mice were intratumorally injected with the FP-Pdots solution (10 μL, 2 mg mL^–1^) without being deoxygenized. For the purpose of comparison, the mice were subcutaneously injected with the FP-Pdots probe under the same conditions. As shown in Fig. 6A, a very intense signal from the area of the tumor (ROI 1) was detected. When compared to the area of the subcutaneous injection (ROI 2), an obviously high signal-to-noise ratio in the emission intensity within the tumor area can be observed. To intuitively estimate the change in luminescence intensity, imaging dynamic curves were plotted, as shown in Fig. 6B. The luminescence intensity of the FP-Pdots located in tumor ROI 1 increased abruptly following 3 seconds of injection into the tumor and subsequently increased. The intensity was up to 3.3 times greater than that at 660 ± 13 nm of the FP-Pdots (ROI 3) compared with the phosphorescence intensity at ROI 2. Therefore, the FP-Pdots exhibited excellent oxygen-sensitivity and can be applied as tumor hypoxia probes *in vivo*.

Lastly, the cytotoxicity of the FP-Pdots nanoprobe toward HepG2 cells was determined *via* an MTT 3-(4,5-dimethylthiazol-2-yl)-2,5-diphenyltetrazolium bromide assay. The plot of cellular viabilities (%) *vs.* incubation concentrations (0–50 μg mL^–1^) of the probe in PBS buffer at 37 °C for 24 h is illustrated in Fig. S8.† The viabilities of the HepG2 cells still retained a value higher than 70% even after incubation with a high concentration (50 μg mL^–1^) of the nanoprobe. Under the living cell imaging experimental conditions (10 μg mL^–1^ concentration, 2 h incubation time), the FP-Pdots nanoprobe showed negligible cytotoxicity toward HepG2 cells with cell viabilities of more than 95%. These data demonstrated that the use of the FP-Pdots as a hypoxia probe shows excellent biocompatibility.

## Conclusions

In conclusion, we have presented a hypoxia nanoprobe based on fluorescent/phosphorescent dual-emissive FP-Pdots, which was prepared from conjugated polyelectrolyte by using polyfluorenes as an O_2_-insensitive fluorophore and Pt(ii) porphyrins as an O_2_-sensitive phosphor. The FP-Pdots nanoprobe was demonstrated to be a kind of excellent ratiometric O_2_ nanoprobe with full reversibility and low toxicity toward living cells. Importantly, the FP-Pdots can perform excellently in PLIM and TGLI techniques, which will evidently improve the sensing sensitivity and reliability. In addition, the FP-Pdots nanoprobe was applied to tumor-bearing mice for luminescence imaging of tumor hypoxia. These initial studies showed that the fluorescent/phosphorescent dual-emissive FP-Pdots are a promising kind of hypoxia nanoprobe, which will be helpful in designing a ratiometric and time-resolved luminescent probe for monitoring tumor hypoxia.
