# Supplementary material for: Fluorescent/phosphorescent dual-emissive conjugated polymer dots for hypoxia bioimaging
Source: Chem Sci. 2015 Jan 12;6(3):1825–31. doi: 10.1039/c4sc03062a (PMC5485888; doi:10.1039/c4sc03062a)
Supplement: Supplementary file 1 [file SC-006-C4SC03062A-s001.pdf]

Electronic Supplementary Material (ESI) for Chemical Science.
This journal is © The Royal Society of Chemistry 2015

Supporting Information

Fluorescent/phosphorescent dual-emissive conjugated polymer dots for hypoxia bioimaging

Qiang Zhao,*[a] Xiaobo Zhou,[a] Tianye Cao,[c] Kenneth Yin Zhang,[a] Lijuan Yang,[a] Shujuan Liu,[a] Hua Liang,[a] Huiran Yang,[a] Fuyou Li,*[c] and Wei Huang*[a,b]

[a] Key Laboratory for Organic Electronics and Information Displays & Institute of Advanced Materials(IAM), Jiangsu National Synergistic Innovation Center for Advanced Materials (SICAM), Nanjing University of Posts & Telecommunications, 9 Wenyuan Road, Nanjing 210023, China. E-mail: iamqzhao@njupt.edu.cn (Q. Zhao); wei-huang@njtech.edu.cn (W. Huang); Fax: (+86) 25-85866396.

[b] Key Laboratory of Flexible Electronics (KLOFE) & Institute of Advanced Materials (IAM), Jiangsu National Synergistic Innovation Center for Advanced Materials (SICAM), Nanjing Tech University (NanjingTech), 30 South Puzhu Road, Nanjing 211816, China.

[c] Department of Chemistry & State Key Laboratory of Molecular Engineering of Polymers & Institute of Biomedicine Science, Fudan University, Shanghai 200433, China. E-mail: fyli@fudan.edu.cn; Fax: (+86) 21-55664621.

General methods

All air and moisture sensitive reactions were carried out under an argon atmosphere. The ^{1}H and ^{13}C NMR spectra were measured at room temperature by using a Bruker Ultra Shield Plus 400 MHz NMR instrument. $CDCl_3$ was used as deuterated reagent unless specified. Mass spectra were measured with a Brukerautoflex MALDI-TOF/TOF mass spectrometer. The UV-visible absorption spectra were obtained with a Shimadzu UV-3600 UV-VIS-NIR spectrophotometer. Photoluminescent spectra were measured using a RF-5301PC spectrofluorophotometer. Emission lifetime studies were performed with an Edinburgh FL920 photocounting system with a semiconductor laser as the excitation source. Transmission electron microscopy (TEM) was conducted on a JEOL transmission electron microscope (JEM-2100) at an acceleration voltage of 100 kV. The number-average molecular weight (M_n) and weight-average molecular weight (M_w) of the polymers were characterized in THF by gel permeation chromatography at 35 °C (polystyrene as standard). For oxygen sensing, Mass Flow Controller (HORIBA, SEC-E40) was used to control

the O_2 concentrations. Standard gas mixtures containing 0, 2, 4, 10, 12, 16, 18, 21, 50, 80, and 100% of O_2 balanced with N_2 (100, 98, 96, 90, 88, 84, 82, 79, 50, 20, and 0%, respectively) were passed through the cuvette for 10 min to equilibrate the oxygen content to the respective concentrations and to monitor the changes of sensor luminescence. The quantum yield for $^1O_2$ photosensitization of FP-Pdots has been measured to be 82% according to the reported method using 2,2'-(anthracene-9,10-diylbis(methylene)) dimalonic acid (ADMA) as the indicator and $[Ru(bpy)_3]^{2+}$ ($\phi_\Delta = 0.81$) as a standard in methanol.[1]

Synthesis

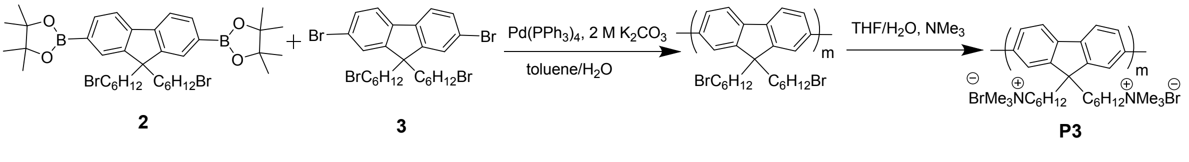

Scheme S1. Synthetic route of the conjugated Pt(II)-free polyelectrolyte **P3**.

Monomer **2** and **3** were synthesized according to the previous report.[2]

5-(Pentafluorophenyl)dipyrromethane: Pyrrole (25.0 mL, 360 mmol) and 2,3,4,5,6-pentafluorobenzaldehyde (5.3 g, 27.2 mmol) were added to a 100 mL dry round-bottomed flask. The solution was degassed with a stream of argon for 20 min. Trifluoroacetic acid (210.0 uL, 2.7 mmol) was added, and the mixture was stirred under argon and dark at room temperature for 1.5 h. The mixture turned yellow during the course of the reaction. Then NaOH (0.1 M, 20 mL) was added to quench the reaction. Stirring for 3 h to make sure that the reaction quenched absolutely, afforded a blue mixture. 100 mL ethyl acetate was then added. The organic phase was washed with 4×80 mL of water and then dried (Na_2SO_4). The crude product obtained after removal of solvents was purified by column chromatography [silica, hexanes/CH_2Cl_2/ethyl acetate (10:2:1)]. It was dissolved in CH_2Cl_2 and then silica gel was added, and the solvent was evaporated. The resulting powder was loaded on the top of the column. Elution (400-1400 mL of eluant) followed by concentration of the eluted product gave a white solid (6.56 g, 77% yield). ^{1}H NMR (400 MHz, $CDCl_3$, δ): 8.16 (br s, 2H), 6.75−6.72 (m, 2H), 6.17 (q, 2H), 6.03 (m, 2H), 5.90 (s, 1H); GC-MS: calcd for $C_{15}H_9N_2F_5$ 312.24, Found 312.32.

5,15-Bis(pentafluorophenyl)-10,20-bis(4-bromophenyl)porphyrin: 4-Bromo-benzaldehyde (583.0 mg, 3.2 mmol) and 5-(pentafluorophenyl)dipyrromethane (1.0 g, 3.2 mmol) were added to a dry round-bottomed 500 mL flask with 320 mL of CH_2Cl_2 and degassed with a stream of

argon for 10 min. BF_3•O(Et)$_2$ (0.3 mL) was then added, the solution was stirred under argon at room temperature for 1 h, and then 2,3-dichloro-5,6-dicyano-1,4-benzoquinone (DDQ, 572.0 mg, 2.5 mmol) was added. The mixture was stirred at room temperature for an additional 1 h, and then the solvent was removed. Column chromatography with elution by CH_2Cl_2/hexane 1:10 (v/v) afforded the porphyrin as purple solid (0.9 g, 20% yield). ^{1}H NMR (400 MHz, $CDCl_3$, *δ*): 8.936 (d, *J*= 4.8 Hz, 2H), 8.868 (dd, *J*=5.6 Hz, 4H), 8.802 (d, *J*=4.8 Hz, 2H), 8.077 (m, *J*=8 Hz, 4H), 7.929 (m, *J*=8 Hz, 4H), −2.83 (br s, 2H). MS (MALDI-TOF) *m/z*: calcd for $C_{44}H_{18}N_4F_{10}Br_2$ 952.43, Found: 953.10.

***Platinum(II) 5,15-Bis(pentafluorophenyl)-10,20-bis(4-bromophenyl)porphyrin*:** K_2PtCl_4 (93.7 mg, 0.23 mmol) and 5,15-bis(pentafluorophenyl)-10,20-bis (4-bromophenyl)-porphyrin (48 mg, 0.05 mmol) were added to a dry round-bottomed 25 mL flask with 7 mL of anhydrous benzonitrile and degassed with a stream of argon for 10 min. The solution was stirred and refluxed under argon at 180 °C for 2 days, the mixture was cooled, and the solvent was removed under vacuum. The crude product was purified with column chromatography on silica gel with CH_2Cl_2/hexane (1:5) as the eluent. Recrystallization from CH_2Cl_2/hexane gave Pt(II) porphyrin complex as brown-red crystals (30 mg, 52%). ^{1}H NMR (400 MHz, $CDCl_3$, *δ*) 8.840 (d, *J*=5.2 Hz, 4H), 8.725 (d, *J*=5.2 Hz, 4H), 8.030 (m, *J* =8.4 Hz, 4H), 7.910 (m, *J* = 8.4 Hz, 4H). MS (MALDI-TOF) *m/z*: calcd for $C_{44}H_{16}N_4F_{10}Br_2Pt$ 1145.34, Found: 1146.37

***P1*:** The mixture of monomer 2,7-dibromo-9,9-bis(6'-bromohexyl)fluorene compound **3** (52 mg, 0.08 mmol), 2,7-(9,9-bis(11-methyloxyl-3,6,9-trioxahendecyl))dibromofluorene compound **2** (74.5 mg, 0.1 mmol) and porphyrin Pt (II) compound **1** (23 mg, 0.02 mmol) in 2.5 mL of toluene and 1.6 mL of 2.0 M potassium carbonate aqueous solution was degassed, and then catalytic quantity of $Pd(PPh_3)_4$ and tetrabutylammoniumbromide (TBAB) was added. The mixture was vigorously stirred at 85 °C under a argon atmosphere for 2 days. After cooling down to room temperature, water (20 mL) was added and the mixture was extracted with CH_2Cl_2. The organic layer was dried over anhydrous $MgSO_4$ and the solvent was removed. The crude product was dissolved in THF (0.5 mL) and then precipitated in CH_3OH / H_2O (10:1). The precipitate was collected by filtration. The precipitate was dissolved in THF and reprecipitated in methanol (200 mL). The precipitate was filtered and washed with acetone and then dried in vacuum for 24 h to afford desired polymer (70 mg, 64%) as an red solid. ^{1}H NMR (400 MHz, $CDCl_3$, *δ*): 8.72-9.03 (m, 0.6H pyrrole H of porphyrin), 8.05-8.40 (m, 0.6H Ar H of porphyrin), 7.60-7.93 (m, 6H, Ar H of fluorene) 3.26 (t, 4H −CH_2Br of fluorene), 2.17 (m, 4H −CH_2 of fluorene) 1.70 (m, 4H −CH_2 of fluorene), 1.00-1.50 (m, −CH_2 of fluorene 8H), 0.80 (m, −CH_2 of fluorene 4H); ^{13}C NMR (100 Hz, $CDCl_3$, *δ*): 151.51, 140.52, 140.12, 127.23, 127.21,

126.33, 121.34, 120.18, 55.34, 40.32, 34.04, 32.63, 29.09, 27.73, 23.72; GPC (THF, polystyrene standard): M_w 63200 g/mol, M_n 25400 g/mol, PDI 2.49.

P2**:** Condensed trimethylamine (2 mL) was added dropwise to a solution of the neutral polymer **P1** (60 mg) in THF (10 mL) at -78 °C using a dry ice-acetone bath. Then the mixture was allowed to warm up to room temperature. The precipitate was re-dissolved by the addition of water (10 mL). After the mixture was cooled down to -78 °C, extra trimethylamine (2 mL) was added and the mixture was stirred for 24 h at room temperature. After removing most of the solvent, acetone was added to precipitate **P2** (62 mg, 89%) as an red powder. ^{1}H NMR (400 MHz, CD_3OD, δ): 8.75-9.05 (m, 0.6H), 8.05-8.40 (m, 0.6H), 7.65-7.95 (m, 6H), 3.30 (t, 4H), 3.08 (s, 17H), 2.29 (br, 4H), 1.60 (br, 4H), 1.00-1.50 (br, 8H), 0.80 (br, 4H). ^{13}C NMR (100 MHz, CD_3OD, δ): 151.8, 140.9, 140.4, 140.0, 127.6, 126.1, 121.2, 120.5, 66.7, 55.7, 52.5, 40.2, 29.2, 25.8, 23.7, 22.5; Quarternization degree: 95%.

Polymer dots (Pdots)[3]**:** 1.2 mg **P2** was dissolved in 5.0 mL methanol by stirring overnight under inert atmosphere. Firstly, 2 mL of **P2** solutions was injected quickly into 8 mL Phosphate Buffered Saline (PBS) while sonicating the mixture to aid mixing. Then, the resulting nanoparticle suspension was filtered through a 0.2 micron membrane filter in order to remove lager aggregates. The methanol was removed by evaporation under vacuum at 38 °C. Additional filteration step was carried out to acquire Pdots.

Calculation of the Förster radius (R_0):

The Förster radius was calculated as follows: $$R_0^6 = \frac{9k^2\varphi_d J(\lambda)}{128N\pi^5 n^4}$$

Where k^2 is the orientation factor of the interacting dipoles (set as 2/3), φ_d the quantum yield of the donor, n the refractive index of the medium separating donor and acceptor chromophore (1.8), $J(\lambda)$ the overlap integral and N the Avogadro's constant.

Confocal luminescence imaging

Confocal luminescence imaging was carried out on an Olympus IX81 laser scanning confocal microscope equipped with a 40 immersion objective len. A semiconductor laser at 405 nm was served as excitation of the HepG2 cells incubated with FP-Pdots nanoprobes. FP-Pdots nanoprobes was added to RPMI 1640 to yield 10 μg/mL solution. The HepG2 cells were incubated with the FP-Pdots (10 μg/mL) nanoprobes for at 37 °C 2 h at 21% or 2.5% O_2 conditions. Then the cells were

immediately transferred into Live Cell Imaging System (OLYMPUS, Xcellence) for confocal luminescence imaging. The emission was collected at 420-460 nm and 630-680 nm for the HepG2 cells incubated with FP-Pdots nanoprobes. The data was shown in Figure 3.

Photoluminescence lifetime imaging

The HepG2 cells were incubated with the FP-Pdots nanoprobe (10 μg/mL) at 37 °C for 2 h at 21% and 2.5% O_2 concentrations, respectively. The PLIM setup is integrated with Olympus IX81 laser scanning confocal microscope. The luminescence signals were detected by confocal microscope system and the correlative calculation of the data was performed using professional software which was provided by PicoQuant GmbH. The excitation light of 405 nm with a frequency of 0.5 MHz from the pulse diode laser (PicoQuant, PDL 800-D) was focused onto the sample with a 40 X objective lens (NA 0.95) for single-photon excitation. The luminescence signals were collected in the range of 420-680 nm. The images were shown in Figure 4.

Time-gated luminescence intensity imaging

The HepG2 cells were incubated with the FP-Pdots nanoprobe (10 μg/mL) at 37 °C for 2 h at 21% and 2.5% O_2 concentrations, respectively. The time-gated images were captured by the professional software provided by PicoQuant GmbH. First, the imaging mode was set to the "Events", and the images containing the intensity signal only were obtained. Then using 250 ns as the gated-time, the image which leaved the signal of the first 250 ns out was captured. Similarly, the images with 500 ns or 1000 ns as the gated-time were also captured through the similar method. The luminescence signals were collected in the range of 420-680 nm as shown in Figure 5.

Cytotoxicity test

The in vitro cytotoxicity was measured using a standard methyl thiazolyl tetrazolium (MTT, Sigma Aldrich) assay in HepG-2 cell lines. Briefly, cells growing in log phase were seeded into 96-well cell culture plate at 1×10^4/well. FP-Pdots nanoprobes was added to the wells of the treatment group at concentrations of 10, 20, 30 and 50 μg/mL. For the negative control group, 1 μL/well solvent was diluted in RPMI 1640 with the final concentration of 1 %. The cells were incubated for 24 h at 37 °C under 5 % CO_2. The combined MTT/PBS solution was added to each well of the 96-well assay plate and incubated for an additional 4 h. After removal of the culture solution, 200 μL DMSO was added to each well, shaking for 10 min at shaking table. An enzyme-linked immunosorbent assay

(ELISA) reader was used to measure the OD570 (absorbance value) of each well referenced at 490 nm. The following formula was used to calculate the viability of cell growth:

Viability (%) = (mean of absorbance value of treatment group / mean of absorbance value of control) × 100

Tumor Xenografts

Animal experiments were conducted according to the guidelines of the Institutional Animal Care and Use Committee. Tumor cells were harvested when they reached near confluence by incubation with 0.05% trypsin-EDTA. Cells were pelleted by centrifugation and resuspended in sterile PBS. The KB cells (5×10^6 cells/site) were implanted subcutaneously into the six-week-old female athymic nude mice. When the tumors reached 0.8 cm in diameter (~ three weeks after implantation), the tumor-bearing mice were subjected to imaging studies.

In Vivo Imaging of Tumors

In vivo luminescence imaging was performed using a modified Kodak *in vivo* imaging system. The Xenon lamp (100 W) was used as the excited source, collocating with a bandpass filter (410 ± 15 nm). The luminescence signals were collected at 660 ± 13 nm, and an Andor DU897 EMCCD as the signal collector. The mice were intratumorally injected with the FP-Pdots solution (10 µL, 2 mg/mL) without being deoxygenized. For the purpose of comparison, the mice were subcutaneously injected with the FP-Pdots probe at the same condition. Images of luminescent signals were analyzed with Kodak Molecular Imaging Software.

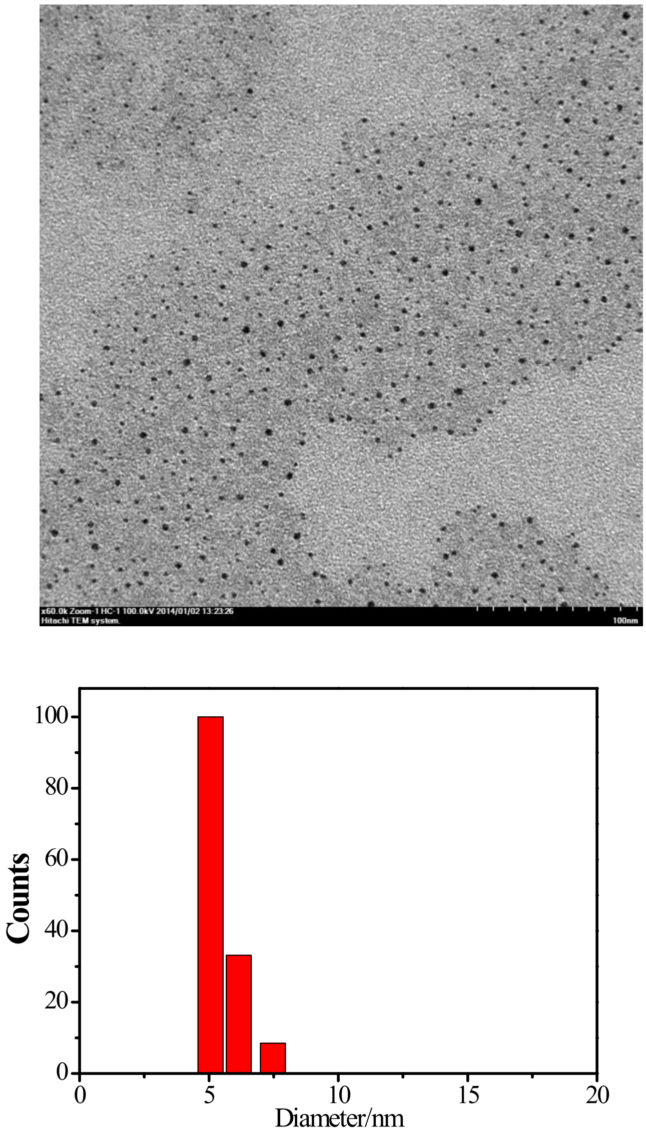

Fig S1. TEM image (top) and DLS (bottom) of FP-Pdots in PBS.

Photophysical properties

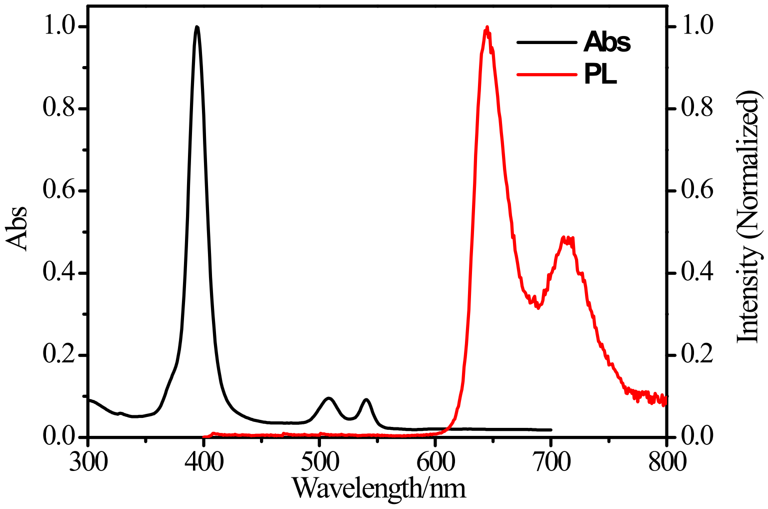

Fig S2. Normalized absorption and emission (λ_{ex} = 395 nm) spectra of Pt(II) porphyrin (**1**, 5.6 μM).

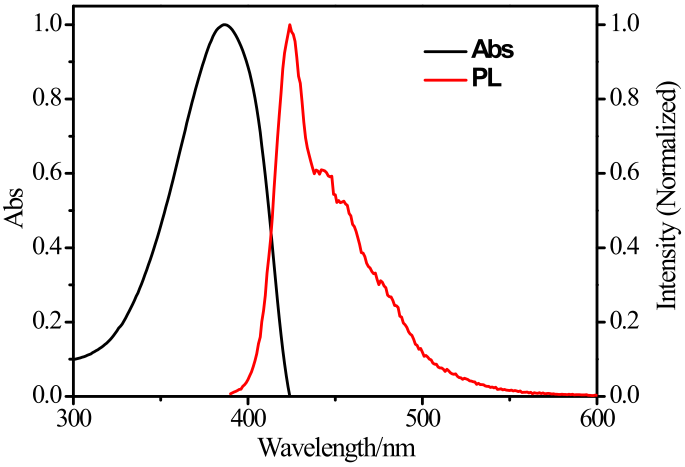

Fig S3. Normalized absorption and emission spectra (λ_{ex} = 375 nm) of Pt(II)-free polymer **P3** (14 μg/mL).

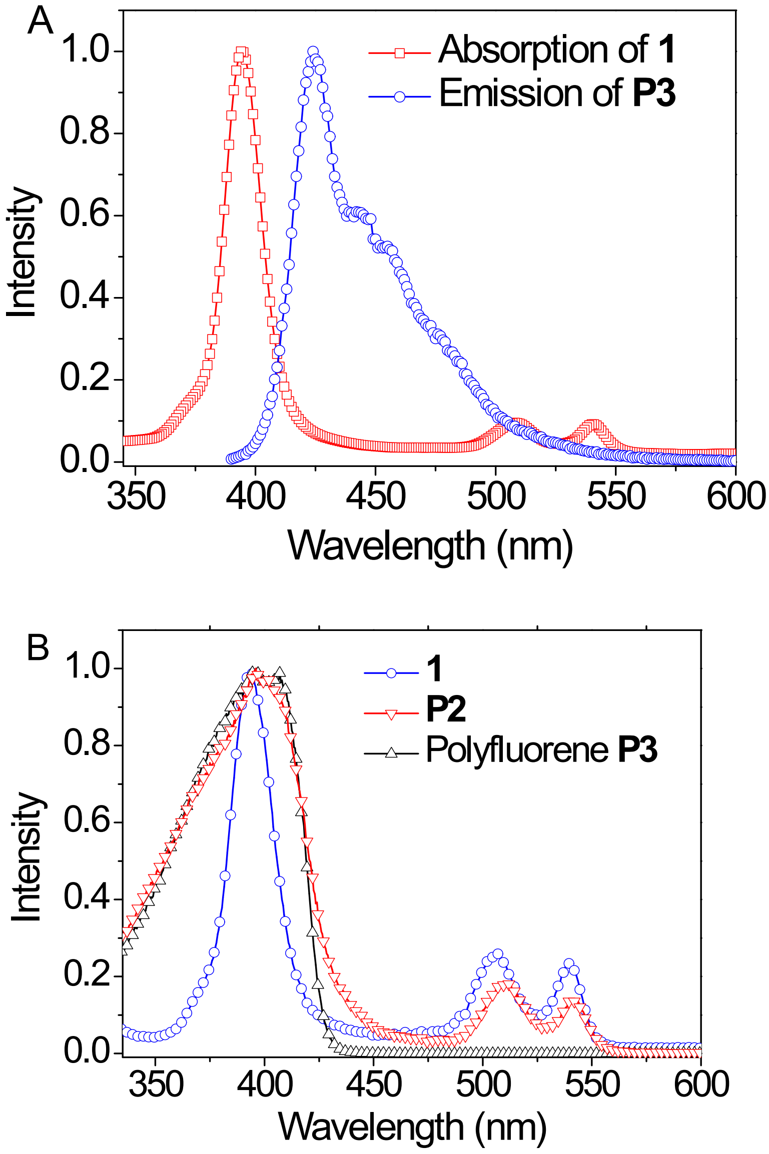

Fig S4. A) Absorption spectrum of Pt(II) porphyrin (**1**, 5.6 μM) and emission (λ_{ex} = 375 nm) spectrum of Pt(II)-free polyfluorene **P3** (14 μg/mL) without platinum(II) porphyrin unit; B) Excitation spectra of Pt(II) porphyrin (**1**) and Pt(II) complex-polymer **P2** (12 μg/mL) monitored at 650 nm, and excitation spectrum of Pt(II)-free polyfluorene **P3** monitored at 420 nm.

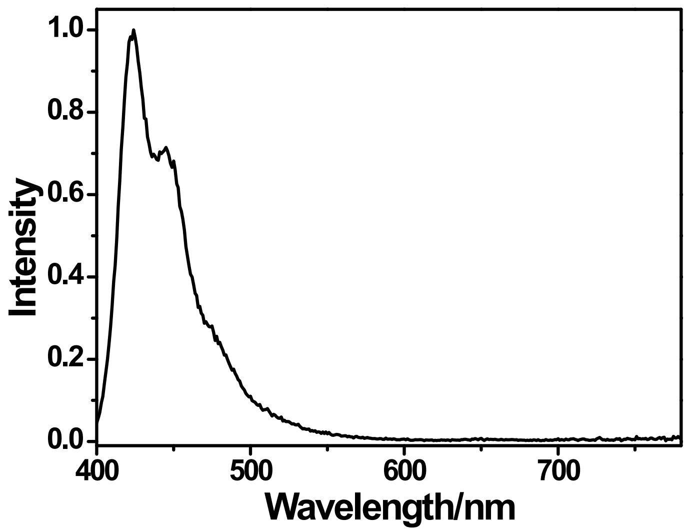

Fig S5. Emission spectrum of the blend of 1.4 μg/mL (1.23 μM) Pt(II) porphyrin (**1**) and 11.5 μg/mL (18.85 μM, equvilent concerntration of fluorine units) Pt(II)-free polyfluorene **P3** in aqueous solution. The molar ratio of Pt: fluorene was calculated as 6.5 : 93.5. (λ_{ex} = 375 nm).

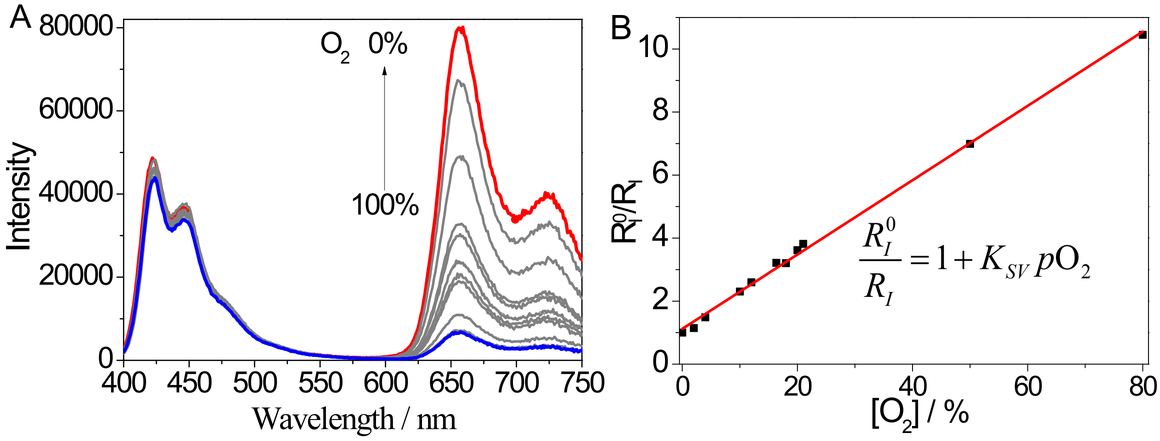

Fig S6. A) Emission spectra of FP-Pdots (12 μg/mL) in H_2O under the different O_2 concentrations; B) Plot of R_I^0/R_I as a function of O_2 concentration(λ_{ex} = 375 nm).

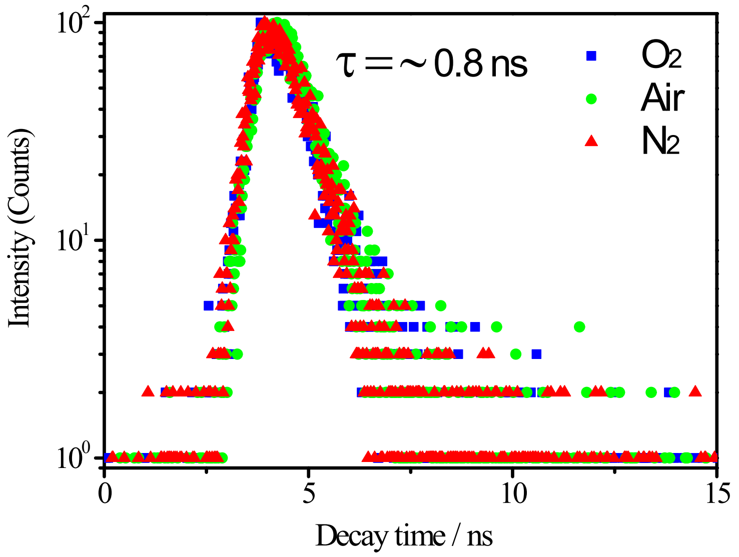

Fig S7. Fluorescence decay curves of FP-Pdots (λ_{ex} = 375 nm, monitored at 425 nm from polyfluorene) in aqueous solution saturated with N_2, air and O_2, respectively. The temporal resolution for the transient photoluminescence experiments is 200 ps.

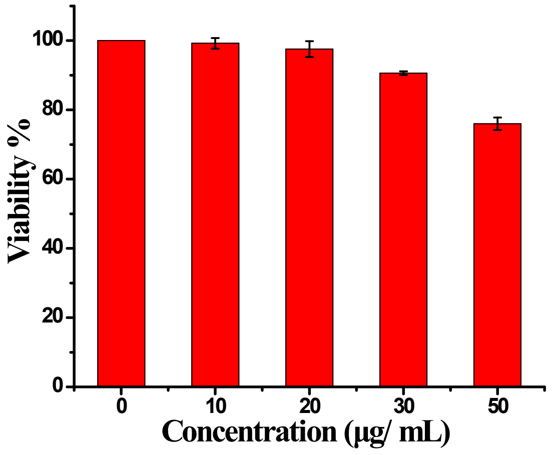

Fig S8. *In vitro* cell viability of HepG2 cells incubated with FP-Pdots at different concentrations for 24 h.

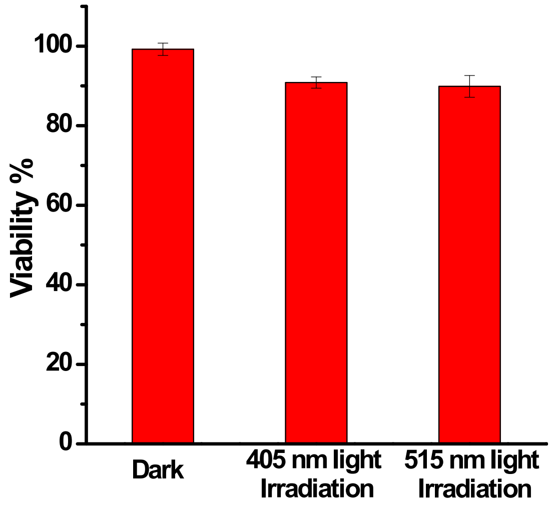

Fig S9. Cell viability of HpeG2 incubated with FP-Pdots (10 μg/mL) by using typical MTT assay under different wavelength light irradiation for 1 min. The irradiation power of 405 nm (generated by semiconductor laser) and 515 nm (generated by multiline Ar laser) light both are 5 mW/cm^2.

REFERENCES

1 B. A. Albani, B. Peña, N. A. Leed, N. A. B. G. de Paula, C. Pavani, M. S. Baptista, K. R. Dunbar, and C. Turro, *J. Am. Chem. Soc.*, 2014, **136**, 17095.

2 H. F. Shi, H. B. Sun, H. R. Yang, S. J. Liu, G. Jenkins, W. Feng, F. Y. Li, Q. Zhao, B. Liu and W. Huang, *Adv. Funct. Mater.*, 2013, **23**, 3268.

3 (*a*) C. Wu and D. T. Chiu, *Angew. Chem. Int. Ed.*, 2013, **52**, 3086; (*b*) C. Wu, C. Szymanski, Z. Cain and J. McNeill, *J. Am. Chem. Soc.*, 2007, **129**, 12904.
